# Supplementary material for: Discovery and application of insertion-deletion (INDEL) polymorphisms for QTL mapping of early life-history traits in Atlantic salmon
Source: BMC Genomics. 2010 Mar 8;11:156. doi: 10.1186/1471-2164-11-156 (PMC2838853; doi:10.1186/1471-2164-11-156)
Supplement: Additional file 2 — Information on developed 76 locus single-run INDEL panel in Atlantic salmon. Information on fluorescence labeling, primer concentrations, PCR pooling and links to alignments, INDEL motifs and GENESCAN (Burge and Karlin 1997) predictions of genes/exons are available in html format. [file 1471-2164-11-156-S2.ZIP › Additionalfile2/snpsummary2136.html]

```
Cluster 366 Contig 4

prev  Summary    Contig List  next
```

Size of Consensus sequence = 1533

Number of sequences = 57

Minimum redundancy = 6

Key

A gi|54021211|gb|CV429002.1|CV429002 EST02231 Atlantic salmon Lambda Zap Express heart cDNA library Salmo salar cDNA clone hr\_002\_e01 5', mRNA sequence  
B gi|117481211|gb|EG813428.1|EG813428 EST\_ssal\_evd\_33406 ssalevd thymus Salmo salar cDNA Salmo salar cDNA clone ssal\_evd\_544\_184\_rev 5', mRNA sequence  
C gi|117471664|gb|EG803883.1|EG803883 EST\_ssal\_evd\_1013 ssalevd thymus Salmo salar cDNA Salmo salar cDNA clone ssal\_evd\_005\_257\_rev 5', mRNA sequence  
D gi|117471665|gb|EG803884.1|EG803884 EST\_ssal\_evd\_1014 ssalevd thymus Salmo salar cDNA Salmo salar cDNA clone ssal\_evd\_005\_257\_fwd 3', mRNA sequence  
E gi|24352901|gb|CA046731.1|CA046731 ssalpitl502252 head Salmo salar cDNA, mRNA sequence  
F gi|117563640|gb|EG884616.1|EG884616 EST\_ssal\_evf\_43056 ssalevf mixed\_tissue Salmo salar cDNA Salmo salar cDNA clone ssal\_evf\_557\_208\_fwd 3', mRNA sequence  
G gi|117481212|gb|EG813429.1|EG813429 EST\_ssal\_evd\_33407 ssalevd thymus Salmo salar cDNA Salmo salar cDNA clone ssal\_evd\_544\_184\_fwd 3', mRNA sequence  
H gi|117859637|gb|EG932333.1|EG932333 EST\_ssal\_evf\_34257 ssalevf mixed\_tissue Salmo salar cDNA Salmo salar cDNA clone ssal\_evf\_545\_221\_fwd 3', mRNA sequence  
I gi|117859638|gb|EG932334.1|EG932334 EST\_ssal\_evf\_34258 ssalevf mixed\_tissue Salmo salar cDNA Salmo salar cDNA clone ssal\_evf\_545\_221\_rev 5', mRNA sequence  
J gi|84568494|gb|DW340113.1|DW340113 SGP310187 Atlantic salmon Ovaries cDNA library Salmo salar cDNA clone KG4-3323 5', mRNA sequence  
K gi|25999180|gb|CA769925.1|CA769925 ssalrga501196 mixed\_tissue Salmo salar cDNA, mRNA sequence  
L gi|29318156|gb|CB506510.1|CB506510 ssalcna010073 muscle\_skin Salmo salar cDNA, mRNA sequence  
M gi|117472188|gb|EG804407.1|EG804407 EST\_ssal\_evd\_1486 ssalevd thymus Salmo salar cDNA Salmo salar cDNA clone ssal\_evd\_006\_208\_rev 5', mRNA sequence  
N gi|85043174|gb|DW571352.1|DW571352 EST\_ssal\_rgb2\_35771 rgb2 Salmo salar cDNA clone ssal\_rgb2\_557\_303\_fwd 3', mRNA sequence  
O gi|117820638|gb|EG893334.1|EG893334 EST\_ssal\_evf\_50902 ssalevf mixed\_tissue Salmo salar cDNA Salmo salar cDNA clone ssal\_evf\_568\_046\_fwd 3', mRNA sequence  
P gi|117848970|gb|EG921666.1|EG921666 EST\_ssal\_evf\_856 ssalevf mixed\_tissue Salmo salar cDNA Salmo salar cDNA clone ssal\_evf\_005\_096\_fwd 3', mRNA sequence  
Q gi|117820639|gb|EG893335.1|EG893335 EST\_ssal\_evf\_50903 ssalevf mixed\_tissue Salmo salar cDNA Salmo salar cDNA clone ssal\_evf\_568\_046\_rev 5', mRNA sequence  
R gi|89874743|gb|DY730866.1|DY730866 EST\_ssal\_rgb2\_86605 ssalrgb2 mixed\_tissue Salmo salar cDNA Salmo salar cDNA clone ssal\_rgb2\_641\_258\_fwd 3', mRNA sequence  
S gi|117570299|gb|EG891275.1|EG891275 EST\_ssal\_evf\_49049 ssalevf mixed\_tissue Salmo salar cDNA Salmo salar cDNA clone ssal\_evf\_565\_236\_rev 5', mRNA sequence  
T gi|117848969|gb|EG921665.1|EG921665 EST\_ssal\_evf\_855 ssalevf mixed\_tissue Salmo salar cDNA Salmo salar cDNA clone ssal\_evf\_005\_096\_rev 5', mRNA sequence  
U gi|24391459|gb|CA061216.1|CA061216 ssalrga509075 mixed\_tissue Salmo salar cDNA, mRNA sequence  
V gi|117455436|gb|EG787655.1|EG787655 EST\_ssal\_evd\_52289 ssalevd thymus Salmo salar cDNA Salmo salar cDNA clone ssal\_evd\_570\_184\_rev 5', mRNA sequence  
W gi|25999580|gb|CA770325.1|CA770325 ssalrga515375 mixed\_tissue Salmo salar cDNA, mRNA sequence  
X gi|24383753|gb|CA053510.1|CA053510 ssalrgb534190 mixed\_tissue Salmo salar cDNA, mRNA sequence  
Y gi|85042764|gb|DW570942.1|DW570942 EST\_ssal\_rgb2\_35361 rgb2 Salmo salar cDNA clone ssal\_rgb2\_557\_069\_fwd 3', mRNA sequence  
Z gi|117472189|gb|EG804408.1|EG804408 EST\_ssal\_evd\_1487 ssalevd thymus Salmo salar cDNA Salmo salar cDNA clone ssal\_evd\_006\_208\_fwd 3', mRNA sequence  
a gi|89878051|gb|DY734174.1|DY734174 EST\_ssal\_rgb2\_89913 ssalrgb2 mixed\_tissue Salmo salar cDNA Salmo salar cDNA clone ssal\_rgb2\_647\_115\_fwd 3', mRNA sequence  
b gi|117570300|gb|EG891276.1|EG891276 EST\_ssal\_evf\_49050 ssalevf mixed\_tissue Salmo salar cDNA Salmo salar cDNA clone ssal\_evf\_565\_236\_fwd 3', mRNA sequence  
c gi|24385152|gb|CA054909.1|CA054909 ssalrga510018 mixed\_tissue Salmo salar cDNA, mRNA sequence  
d gi|117455325|gb|EG787544.1|EG787544 EST\_ssal\_evd\_52288 ssalevd thymus Salmo salar cDNA Salmo salar cDNA clone ssal\_evd\_570\_184\_fwd 3', mRNA sequence  
e gi|117460112|gb|EG792331.1|EG792331 EST\_ssal\_evd\_12418 ssalevd thymus Salmo salar cDNA Salmo salar cDNA clone ssal\_evd\_515\_181\_rev 5', mRNA sequence  
f gi|117821623|gb|EG894319.1|EG894319 EST\_ssal\_evf\_51788 ssalevf mixed\_tissue Salmo salar cDNA Salmo salar cDNA clone ssal\_evf\_569\_127\_rev 5', mRNA sequence  
g gi|45328900|gb|CK899167.1|CK899167 SGP162755 Atlantic salmon Testis cDNA library Salmo salar cDNA clone MG4-1519 5', mRNA sequence  
h gi|117460113|gb|EG792332.1|EG792332 EST\_ssal\_evd\_12419 ssalevd thymus Salmo salar cDNA Salmo salar cDNA clone ssal\_evd\_515\_181\_fwd 3', mRNA sequence  
i gi|45315074|gb|CK885443.1|CK885443 SGP165816 Atlantic salmon Intestine cDNA library Salmo salar cDNA clone T4-1469 5', mRNA sequence  
j gi|117821622|gb|EG894318.1|EG894318 EST\_ssal\_evf\_51787 ssalevf mixed\_tissue Salmo salar cDNA Salmo salar cDNA clone ssal\_evf\_569\_127\_fwd 3', mRNA sequence  
k gi|45309452|gb|CK879821.1|CK879821 SGP144007 Atlantic salmon Gills cDNA library Salmo salar cDNA clone G5-1659 5', mRNA sequence  
l gi|45318044|gb|CK888413.1|CK888413 SGP149623 Atlantic salmon Liver cDNA library Salmo salar cDNA clone L5-0680 5', mRNA sequence  
m gi|40541724|gb|BM413986.1|BM413986 EST00908 Atlantic salmon Lambda Zap Express ovary cDNA library Salmo salar cDNA clone oyr\_003\_g01 5' similar to Annexin max 3, CAA72124, mRNA sequence  
n gi|70786633|gb|DR696273.1|DR696273 SMV3-0017 Atlantic Salmon macrophage - Aeromonas in vivo infection Salmo salar cDNA clone SMV3-0017, mRNA sequence  
o gi|45321161|gb|CK891428.1|CK891428 SGP163690 Atlantic salmon Ovaries cDNA library Salmo salar cDNA clone KG4-1150 5', mRNA sequence  
p gi|85042763|gb|DW570941.1|DW570941 EST\_ssal\_rgb2\_35360 rgb2 Salmo salar cDNA clone ssal\_rgb2\_557\_069\_rev 5', mRNA sequence  
q gi|89874742|gb|DY730865.1|DY730865 EST\_ssal\_rgb2\_86604 ssalrgb2 mixed\_tissue Salmo salar cDNA Salmo salar cDNA clone ssal\_rgb2\_641\_258\_rev 5', mRNA sequence  
r gi|85043173|gb|DW571351.1|DW571351 EST\_ssal\_rgb2\_35770 rgb2 Salmo salar cDNA clone ssal\_rgb2\_557\_303\_rev 5', mRNA sequence  
s gi|85030511|gb|DW559167.1|DW559167 EST\_ssal\_rgb2\_23586 rgb2 Salmo salar cDNA clone ssal\_rgb2\_538\_117\_rev 5', mRNA sequence  
t gi|89856409|gb|DY712532.1|DY712532 EST\_ssal\_rgb2\_68271 ssalrgb2 mixed\_tissue Salmo salar cDNA Salmo salar cDNA clone ssal\_rgb2\_610\_341\_rev 5', mRNA sequence  
u gi|89851015|gb|DY707138.1|DY707138 EST\_ssal\_rgb2\_62877 ssalrgb2 mixed\_tissue Salmo salar cDNA Salmo salar cDNA clone ssal\_rgb2\_601\_211\_rev 5', mRNA sequence  
v gi|89882582|gb|DY738705.1|DY738705 EST\_ssal\_rgb2\_94444 ssalrgb2 mixed\_tissue Salmo salar cDNA Salmo salar cDNA clone ssal\_rgb2\_654\_303\_rev 5', mRNA sequence  
w gi|89878050|gb|DY734173.1|DY734173 EST\_ssal\_rgb2\_89912 ssalrgb2 mixed\_tissue Salmo salar cDNA Salmo salar cDNA clone ssal\_rgb2\_647\_115\_rev 5', mRNA sequence  
x gi|85022744|gb|DW551400.1|DW551400 EST\_ssal\_rgb2\_15819 rgb2 Salmo salar cDNA clone ssal\_rgb2\_526\_071\_rev 5', mRNA sequence  
y gi|85019280|gb|DW547936.1|DW547936 EST\_ssal\_rgb2\_12355 rgb2 Salmo salar cDNA clone ssal\_rgb2\_520\_308\_rev 5', mRNA sequence  
z gi|57122734|gb|CX354175.1|CX354175 ssalrgb534190\_rev\_0 mixed\_tissue Salmo salar cDNA, mRNA sequence  
A gi|57122817|gb|CX354258.1|CX354258 ssalrgb519234\_rev\_0 mixed\_tissue Salmo salar cDNA, mRNA sequence  
B gi|40541614|gb|BM413876.1|BM413876 EST00798 Atlantic salmon Lambda Zap Express testis cDNA library Salmo salar cDNA clone tsr\_004\_b11 5' similar to Annexin max 3, CAA72124, mRNA sequence  
C gi|45312551|gb|CK882920.1|CK882920 SGP147605 Atlantic salmon Heart cDNA library Salmo salar cDNA clone H6-0574 5', mRNA sequence  
D gi|84972561|gb|DW470962.1|DW470962 SGP313594 Atlantic salmon Testis cDNA library Salmo salar cDNA clone MG4-4376 5', mRNA sequence  
E gi|89876576|gb|DY732699.1|DY732699 EST\_ssal\_rgb2\_88438 ssalrgb2 mixed\_tissue Salmo salar cDNA Salmo salar cDNA clone ssal\_rgb2\_644\_228\_rev 5', mRNA sequence

8 SNPs detected

A B C D E F G H I J K L M N O P Q R S T U V W X Y Z a b c d e f g h i j k l m n o p q r s t u v w x y z A B C D E  cosegregation weighted

113 T A A A T T A A A T . T T T A A A T A A T T . T T T T . T . T T . . . . . . . . . . . . . . . . . . . . . . . . .   4/8 24.56
149 T - - - T T - - - T T T T T - - - T - - T T T T T T T . T . T T . T . . . . . . . . . . . . . . . . . . . . . . .   4/8 27.19
150 A - - - A A - - - A A A A A - - - A - - A A A A A A A . A . A A . A . . . . . . . . . . . . . . . . . . . . . . .   4/8 27.19
290 C T T T C C T T T C C C C C T T T C T T C C C C C C C . C C C C . C . C . . . . . . . . . . . . . . . . . . . . .   4/8 28.95
371 . C C C C - C C C C - - - - C C C C C C C - - - - - C C C - - - . - . - . . . . . . . . . . . . . . . . . . . . .   4/8 28.95
372 . T T T T - T T T T - - - - T T T T T T T - - - - - T T T - - - . - . - . . . . . . . . . . . . . . . . . . . . .   4/8 28.95
373 . G G G G - G G G G - - - - G G G G G G G - - - - - G G G - - - . - . - . . . . . . . . . . . . . . . . . . . . .   4/8 28.95
374 . A A A A - A A A A - - - - A A A A A A A - - - - - A A A - - - . - . - . . . . . . . . . . . . . . . . . . . . .   4/8 28.95
